# [Retracted] Combined application of Embelin and tumor necrosis factor-related apoptosis-inducing ligand inhibits proliferation and invasion in osteosarcoma cells via caspase-induced apoptosis

**DOI:** 10.3892/ol.2026.15687

**Published:** 2026-06-08

**Authors:** Hao Qian, Yao Chen, Tao Huang, Tiemin Liu, Xiucheng Li, Guangjian Jiang, Wei Zhang, Shuo Cheng, Pengcheng Li

Oncol Lett 15: 6931–6940, 2018; DOI: 10.3892/ol.2018.8209

Following the publication of the above paper, it was drawn to the Editor's attention by a concerned reader that, regarding the cell invasion assay data shown in Fig. 4 on p. 6936, four of the eight data panels featured in Fig. 4A and B contained overlapping sections, such that data which were intended to show the results from differently performed experiments had been derived from the same original source. Moreover, upon performing an independent analysis of the data in this paper in the Editorial Office, it also came to light that, concerning the cell morphological images shown in Fig. 2A and B and in Fig. 3A and B, two pairs of data panels also contained overlapping sections, comparing betweeen these figures.

Given the large number of overlapping data panels that have been identified in the above paper, suggesting that data were incorrectly assembled in Fig. 2, Fig. 3, 4, the Editor of *Oncology Letters* has decided that it should be retracted from the Journal on account of a lack of confidence in the scientific integrity of the study. The authors were asked for an explanation to account for these concerns, but the Editorial Office did not receive a reply. The Editor apologizes to the readership for any inconvenience caused.

